# Preoperative prediction of residual back pain after vertebral augmentation for osteoporotic vertebral compression fractures: Initial application of a radiomics score based nomogram

**DOI:** 10.3389/fendo.2022.1093508

**Published:** 2022-12-23

**Authors:** Chen Ge, Zhe Chen, Yazhou Lin, Yuehuan Zheng, Peng Cao, Xiaoyong Chen

**Affiliations:** ^1^ Department of Orthopaedics, Ruijin Hospital, Shanghai Jiao Tong University School of Medicine, Shanghai, China; ^2^ Department of Radiology, Ruijin Hospital, Shanghai Jiao Tong University School of Medicine, Shanghai, China

**Keywords:** osteoporotic vertebral compression fracture, vertebral augmentation, residual back pain, radiomics, nomogram

## Abstract

**Background:**

Most patients with osteoporotic vertebral compression fracture (OVCF) obtain pain relief after vertebral augmentation, but some will experience residual back pain (RBP) after surgery. Although several risk factors of RBP have been reported, it is still difficult to estimate the risk of RBP preoperatively. Radiomics is helpful for disease diagnosis and outcome prediction by establishing complementary relationships between human-recognizable and computer-extracted features. However, musculoskeletal radiomics investigations are less frequently reported.

**Objective:**

This study aims to establish a radiomics score (rad-score) based nomogram for the preoperative prediction of RBP in OVCF patients.

**Methods:**

The training cohort of 731 OVCF patients was used for nomogram development, and the validation cohort was utilized for performance test. RBP was determined as the score of visual analogue scale ≥ 4 at both 3 and 30 days following surgery. After normalization, the RBP-related radiomics features were selected to create rad-scores. These rad-scores, along with the RBP predictors initially identified by univariate analyses, were included in the multivariate analysis to establish a nomogram for the assessment of the RBP risk in OVCF patients preoperatively.

**Results:**

A total of 81 patients (11.2%) developed RBP postoperatively. We finally selected 8 radiomics features from 1316 features extracted from each segmented image to determine the rad-score. Multivariate analysis revealed that the rad-score plus bone mineral density, intravertebral cleft, and thoracolumbar fascia injury were independent factors of RBP. Our nomograms based on these factors demonstrated good discrimination, calibration, and clinical utility in both training and validation cohorts. Furthermore, it achieved better performance than the rad-score itself, as well as the nomogram only incorporating regular features.

**Conclusion:**

We developed and validated a nomogram incorporating the rad-score and regular features for preoperative prediction of the RBP risk in OVCF patients, which contributed to improved surgical outcomes and patient satisfaction.

## Introduction

A skeletal disorder called osteoporosis is characterized by diminished bone mass and altered bone microstructure (1). One of the common complications in elderly individuals with osteoporosis is osteoporotic vertebral compression fracture (OVCF), which primarily causes persistent back discomfort, localized kyphosis, and poor quality of life. It has emerged as an important global health issue currently (2, 3). Vertebral augmentation including percutaneous vertebroplasty (PVP) and percutaneous kyphoplasty (PKP) is now well accepted in the treatment of OVCF in terms of pain relief, bone strengthening, and early mobilization (4, 5). Although pain was relieved in most patients, a small but significant proportion of patients experienced residual back pain (RBP) after vertebral augmentation procedures. Unsatisfactory back pain relief has been reported in approximately 5-20%, which indicates that RBP after OVCF treatment is not rare and can negatively impact daily activities (6–9). Previously, several studies have reported a variety of risk variables that are responsible for RBP after both PVP and PKP, including bone mineral density (BMD), thoracolumbar fascia (TLF) injury, intravertebral cleft (IVC), bone cement leakage, etc (10–13). However, it is still challenging to preoperatively predict the risk of RBP. This may make it more difficult for physicians to recognize the analgesic effects of PVP/PKP, as well as poorer patient satisfaction with surgery and potentially diminished physician trust.

Radiomics is a brand new imaging method with the assistance of computer-aided technology that converts medical imaging into mineable data by extracting high-throughput features to quantify the heterogeneity of regions of interest (ROIs) in radiological images (14). The radiomics approach can establish a complementary relationship between human-recognizable and computer-extracted features. Moreover, radiomics along with existing useful indicators can increase the precision of disease diagnosis and outcome prediction (15, 16). If properly leveraged, the radiomics score (rad-score) based on computer tomography (CT) images has the potential to improve RBP risk prediction without additional cost.

Here, we sought to investigate whether bone rad-scores may enhance RBP risk prediction following vertebral augmentation procedures and to establish a rad-score based nomogram to make a preoperative prediction of RBP in OVCF patients. It could help to identify patients with a high likelihood of RBP, allow for appropriate intervention, and improve clinical outcomes while aligning patient expectations prior to surgery.

## Materials and methods

This prospective cohort study was performed in accordance with the Declaration of Helsinki. It received approval from the Ethics Committee of Ruijin Hospital, Shanghai Jiao Tong University School of Medicine (2013–60). Informed consents were given by all patients.

### Patient selection

Between January 2015 and January 2022, a total of 876 patients with single-segment OVCFs who underwent unilateral PKP in our center were enrolled. Patients in the study satisfied the following inclusion criteria: 1) aged > 55 years, 2) first-time PKP procedure, 3) single segment vertebral fracture of T4-L5, 4) thoracolumbar MRI suggesting bone marrow edema (hypointense T1 and hyperintense T2), 5) diagnosed with osteoporosis by dual energy X-ray absorptiometry (T score ≤ -2.5), and 6) obvious thoracolumbar and back pain with a score of six or more on the visual analogue scale (VAS) and limited physical activity. The following were the exclusion criteria: 1) patients with other medical conditions that might cause thoracolumbar and back pain, such as infections, malignant tumors, and a history of spinal surgery, 2) patients whose spinal canal invaded, 3) unable to tolerate surgery, such as when severe cardiopulmonary comorbidity is present, and 4) patients with insufficient follow-up data.

**Figure 1:**
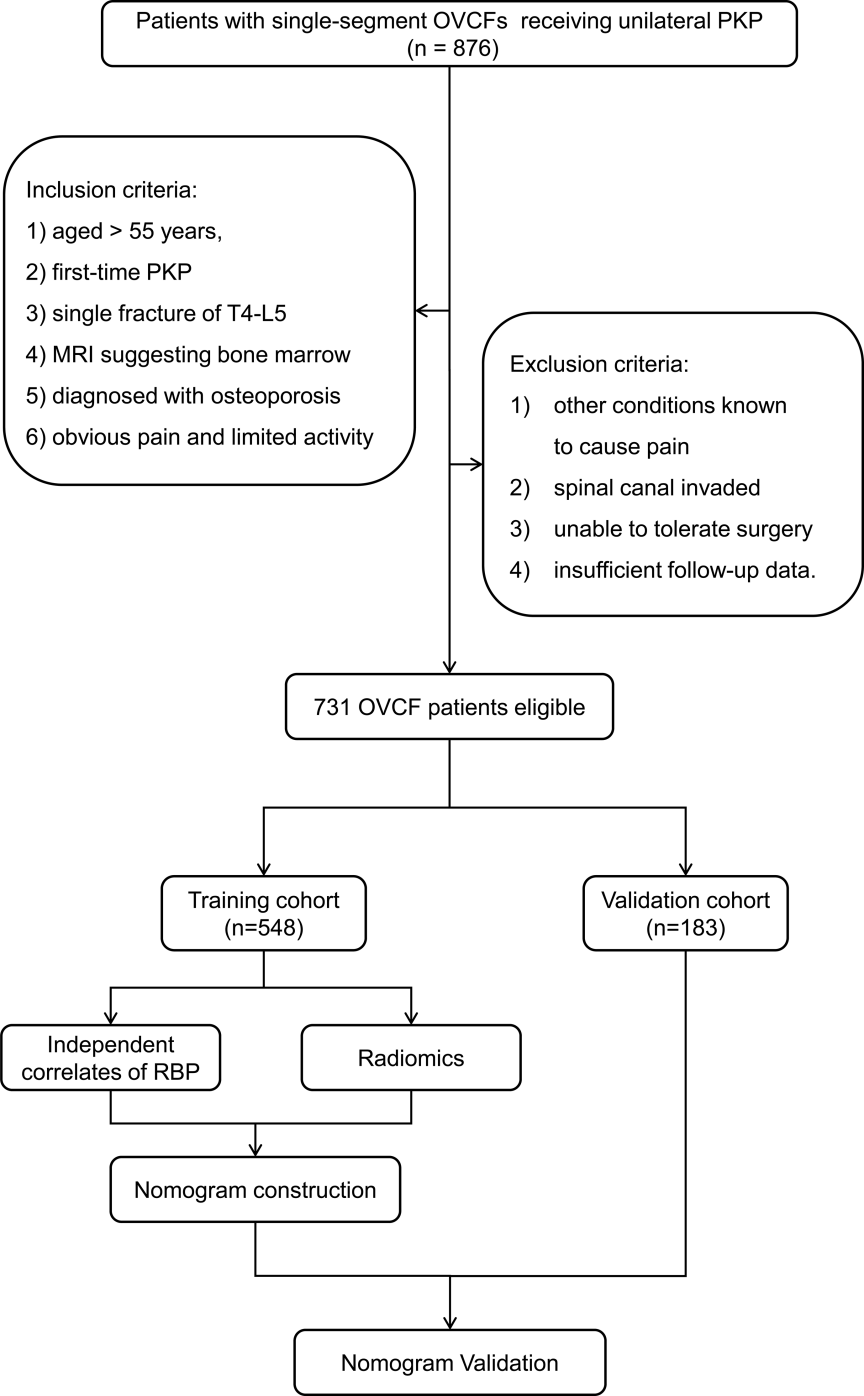
Flowchart of patient selection based on the inclusion and exclusion criteria and model design. OVCF, osteoporotic vertebral compression fracture; PKP, percutaneous kyphoplasty; RBP, residual back pain.

In the end, 731 strictly screened OVCF patients were recruited. Patients between January 2017 and December 2020 were involved in a training cohort (n = 548) and those between January 2021 and January 2022 were included in a validation cohort (n = 183). **Figure 1** shows the flowchart illustrating patient selection and model design.

### Baseline data and radiological parameters collection

The baseline data included age, gender, body mass index (BMI), comorbidities (hypertension, diabetes), smoking status, BMD, fracture position, as well as preoperative VAS score and oswestry disability index (ODI). Radiological parameters, including vertebral height loss, Cobb angle (°) (the angle between the superior and inferior endplate of the fractured level), presence of IVC, and TLF injury were obtained from preoperative X-ray, CT, and MRI imaging.

### Surgical procedures

Patients were placed in the prone position for PKP while being given local or general anaesthetic. A bone puncture trocar was placed at the broken level *via* the lateral pedicle edge and gradually advanced through the pedicle into the vertebral body under C-arm fluoroscopic guidance. The vertebral body was then carefully injected with polymethylmethacrylate using an inflated bone tamp. If cement spilled into extraosseous structures or veins, reached the cortical margin of the vertebral body, or both, the injection was terminated. On the first day following PKP, ambulation was suggested for all patients. Alendronate (70 mg/week), calcitriol (0.25–0.5 g/day), and calcium tablets (600–1200 mg/day) were given postoperatively. Non-steroidal anti-inflammatory medicines were administered to patients with inadequate pain relief or obvious pain that interfered with daily life.

### Study outcome

Patients were followed up and the VAS scores were recorded at four different time intervals, including 1, 3, 7 and 30 days after surgery. RBP was defined as the VAS score ≥ 4 at both 3 and 30 days based on previous experience and literature review due to the lack of existing guidelines (9, 17).

### Image acquisition for radiomics

CT scans were acquired from a 64-slice spiral CT scanner (Aquilion Prime Model TSX-303A, Toshiba Medical Systems Corp., Tokyo, Japan) using the following scanning parameters: tube voltage 120-130 kV, variable mAs to achieve a noise index of 25, rotation time 0.5 or 0.75 s, and matrix 512 × 512. In all cases, axial views with a slice thickness of 3 mm were assessed initially, followed by sagittal and coronal reformations using the inhouse CT postprocessing software.

### Segmentation and feature extraction

**Figure 2:**
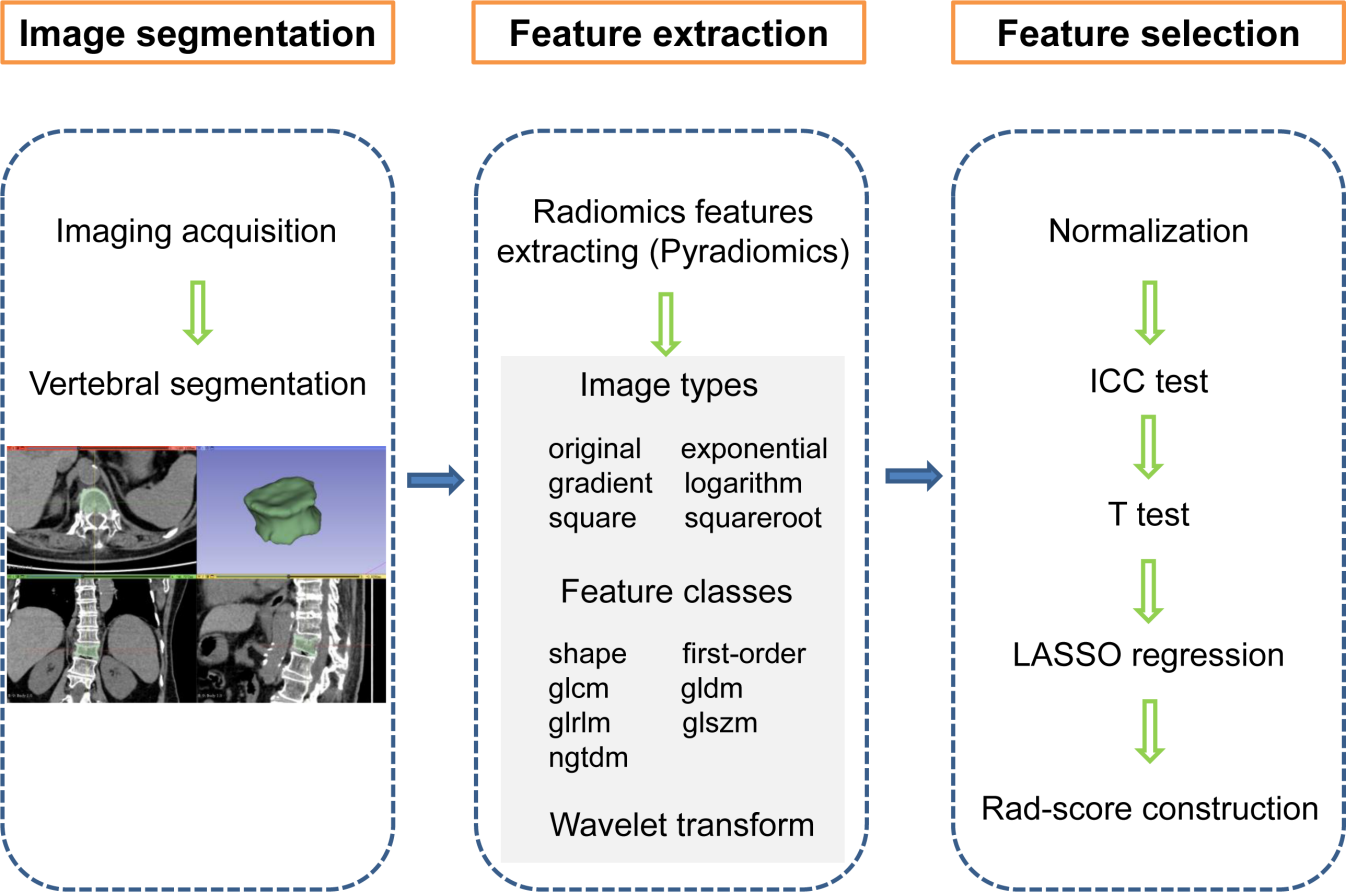
Radiomics workflow showing the construction of a CT imaging-based rad-score for an OVCF patient. The steps include (1) image segmentation, (2) feature extraction, and (3) feature selection. first-order, first order statistics; glcm, gray-level co-occurrence matrix, gldm, gray-level dependence matrix; glrlm, gray-level run length matrix; glszm, gray-level size zone matrix; ngtdm, neighbouring grey tone difference matrix, ICC, intraclass correlation coefficient; LASSO, least absolute shrinkage and selection operator, Rad-score, radiomics score.

The radiomics workflow is shown in **Figure 2**, which mainly includes image segmentation, feature extraction, and feature selection. Vertebral segmentation was performed separately by two radiologists with more than 10 years of clinical experience using 3D Slicer software (v4.10.2) to manually delineate an ROI containing the entire vertebral body slice by slice on axial images. The radiomics features were extracted with the open-source Pyradiomics package. There were 6 image types (original, exponential, gradient, logarithm, square, and squareroot) and 7 feature classes including shape, first-order statistics (first-order), gray-level co-occurrence matrix (glcm), gray-level dependence matrix (gldm), gray-level run length matrix (glrlm), gray-level size zone matrix (glszm), and neighbouring grey tone difference matrix (ngtdm) adopted for each sequence (18). We employed wavelet decomposition filtering after image reconstruction.

### Feature selection and rad-score development

All features extracted from each segmented image were normalized using the standard score (z-score) transformation. To avoid overfitting and improve feature repeatability, three steps were taken in the feature selection process. First, the intraclass correlation coefficient (ICC), with an ICC > 0.9 considered reliable, was used to evaluate discrepancies between the features delineated by two radiologists. Each stable feature was then compared between patient cohorts with and without RBP using a Student t-test. Finally, the features with false discovery rate (FDR)-adjusted P values < 0.05 using Benjamini–Hochberg correction were then subjected to least absolute shrinkage and selection operator (LASSO) regression analysis. Each rad-score is constructed by weighting the coefficients of selected radiomics features based on the output of the LASSO regression.

### Nomogram establishment and validation

The rad-scores and those RBP predictors initially screened by univariate analyses were included in the subsequent multivariate analysis to determine the independent correlates of RBP. These independent variables were then used to create a nomogram for preoperatively identifying whether an OVCF patient will suffer RBP. Prior to being externally evaluated in the validation cohort, its performance was first assessed in the training cohort.

### Statistical analysis

In radiomics, feature extraction, normalization, selection, and rad-score creation were run under Python 3.7.1. Univariate and multivariate Logistic regression analyses were applied to screen for the independent correlates of RBP. The rad-scores plus those RBP predictors that had been first assessed by univariate studies were involved in the subsequent multivariate analysis to determine the odds ratio (OR) and corresponding 95% confidence interval (CI) of each predictor. The nomogram was established according to these correlates and then validated in the training and validation cohorts. Bootstraps analyses were utilized to evaluate the unbiased performance of the model. We evaluated the discrimination and calibration of the nomogram by the Harrell’s concordance index (C-index) combined with the receiver operating characteristic (ROC) curve, and the calibration curve along with the Hosmer-Lemeshow (HL) test. The clinical utility was assessed using a decision curve analysis (DCA), which quantified the net benefit of each decision strategy at each threshold probability. SPSS (version 20.0), MedCalc software (version 19.2.1), and the R package (version 4.2.1) were used for all statistical analyses not related to radiomics.

## Results

### Patient summary

Finally, 731 OVCF patients were enrolled (548 patients in the training cohort for model development and 183 in the validation cohort for evaluating model performance). RBP accounted for 11.2% (81/731) in the overall cohort while 10.8% (59/548) and 12.0% (22/183) in the training and validation cohorts, respectively. There were no statistically significant differences in baseline data and preoperative radiological parameters between the training and validation cohorts (all *P* values > 0.05). The details of two the cohorts are listed in **Supplemental Table 1**.

### Radiomics features analyses for rad-score

**Figure 3:**
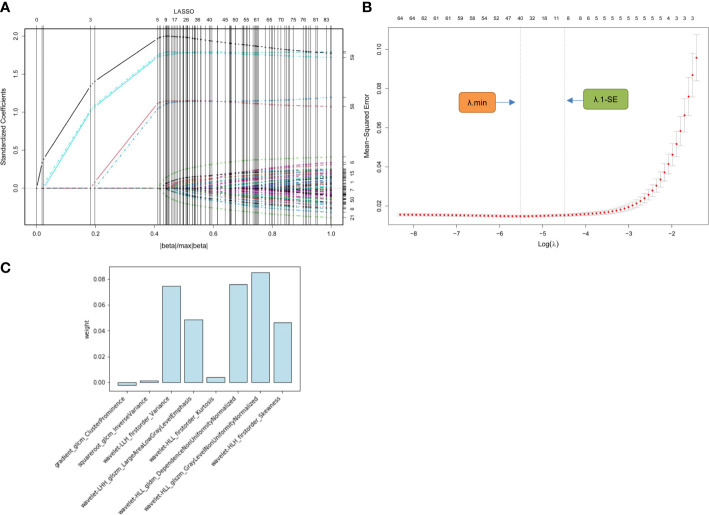
Radiomics feature selection using LASSO regression model. **(A)** Displays the LASSO coefficient profiles of the 85 radiomics features. **(B)** Shows the tuning λ selection in the LASSO regression. Two dotted vertical lines were plotted at the optimal λ values based on the minimum criteria (λ.min) and the 1-standard error for the minimum criteria (λ.1-SE), where the optimal λ resulted in 8 nonzero coefficients. **(C)** Lists the weights of the selected radiomics features. LASSO, least absolute shrinkage and selection operator; λ, penalty regularization parameter.

In the training cohort, 1316 radiomics features for each segmented image were extracted and normalized. Of these, 1077 (81.8%) features with favorable reproducibility (ICC > 0.9) were selected for subsequent t-test screening, resulting in 85 initially screened radiomics features. By constructing a penalty function, 8 radiomics features were chosen by determining the best penalty regularization parameter (λ) with the 1-standard error for the minimum criteria in the LASSO model (**Figure 3**). The rad-score of each patient was established based on the weights of these 8 features and their corresponding coefficients. The calculation of rad-score is shown in **Supplemental Appendix 1**.

### Independent predictors associated with RBP

The results of univariate and multivariate analyses to determine the independent correlates of RBP are summarized in **Table 1**. Rad-score plus BMD, IVC, and TLF injury were incorporated into the multivariate analysis after being filtered by the univariate analyses. The result revealed that RBP was independently associated with BMD, IVC, TLF injury, and rad-score (all *P* < 0.05). RBP was more prevalent in OVCF patients who had IVC, TLF injury, higher rad-score, and lower BMD (OR: 3.220, 11.377, 1.062, and 0.082, respectively).

**Table 1:** Univariate and multivariate Logistic regression analyses for independent correlates of RBP.

Variable		Univariate		Multivariate	
		OR (95% CI)	*P*	OR (95% CI)	*P*
Age		1.019 (0.951-1.091)	0.598		
Gender	Male	Reference			
	Female	0.692 (0.379-1.265)	0.231		
BMI		1.009 (0.906-1.124)	0.873		
Fracture position	T4-T10	Reference			
	T11-L2	1.590 (0.594-4.253)	0.356		
	L3-L5	0.950 (0.339-2.663)	0.922		
Hypertension		1.004 (0.583-1.730)	0.988		
Diabetes		1.209 (0.521-2.807)	0.659		
Smoking		1.074 (0.536-2.152)	0.841		
BMD		0.077 (0.035-0.171)	<0.001	0.082 (0.029-0.232)	<0.001
Preoperative VAS		1.186 (0.903-1.559)	0.220		
Preoperative ODI		1.048 (0.948-1.158)	0.361		
Vertebral height loss		1.013 (0.968-1.059)	0.588		
Cobb angle		1.014 (0.960-1.072)	0.612		
IVC		4.299 (2.143-8.624)	<0.001	3.220 (1.051-9.866)	0.041
TLF injury		6.933 (3.301-14.564)	<0.001	11.377 (4.049-31.962)	<0.001
Rad-score		1.059 (1.045-1.073)^*^	<0.001	1.062 (1.046-1.078)^*^	<0.001

^*^denotes a specific OR value, indicating that the risk increases by 0.01 unit increments. BMI, body mass index; BMD, bone mineral density; VAS, visual analogue scale; ODI, oswestry disability index; Cobb angle, the angle between the superior and inferior endplate of the fractured level; TLF, thoracolumbar fascia; IVC, intravertebral cleft; Rad-score, radiomics score; OR, odds ratio.

### Radiomics Nomogram for RBP

**Figure 4:**
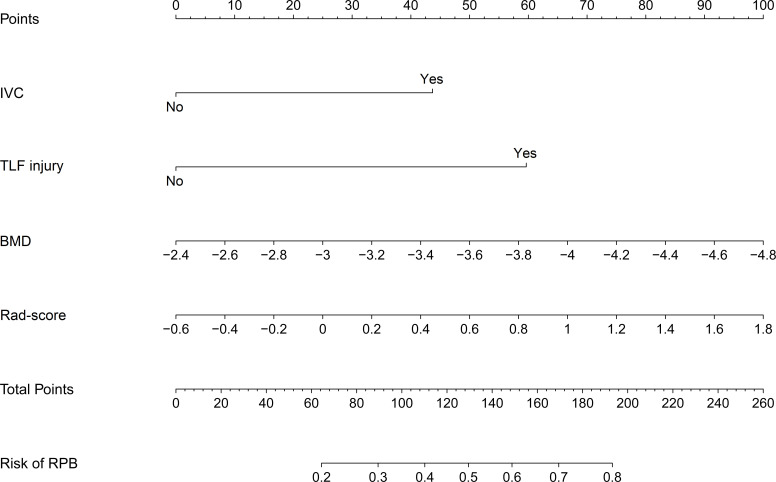
Nomogram for preoperatively assessing the likelihood of encountering RBP in OVCF patients. It is developed with IVC, TLF injury, BMD, and Rad-score with weights equal to the OR values. IVC, intravertebral cleft; TLF, thoracolumbar fascia; BMD, bone mineral density; Rad-score, radiomics score; OR, odds ratio.

Using these selected variables, a radiomics nomogram was developed to preoperatively visualize the likelihood of RBP in OVCF patients. As indicated in **Figure 4**, RBP risk was assessed by accumulating the points corresponding to each variable. For instance, the BMD and rad-score of a 70-year-old female OVCF patient with TLF injury are -3.1 and 0.9, respectively. The corresponding scores are as follows: 0 points for IVC, 60 points for TLF injury, 30 points for BMD, and 60 points for rad-score. She has about a 60% chance of encountering RBP based on her overall score of 150.

### Nomogram validation

**Figure 5:**
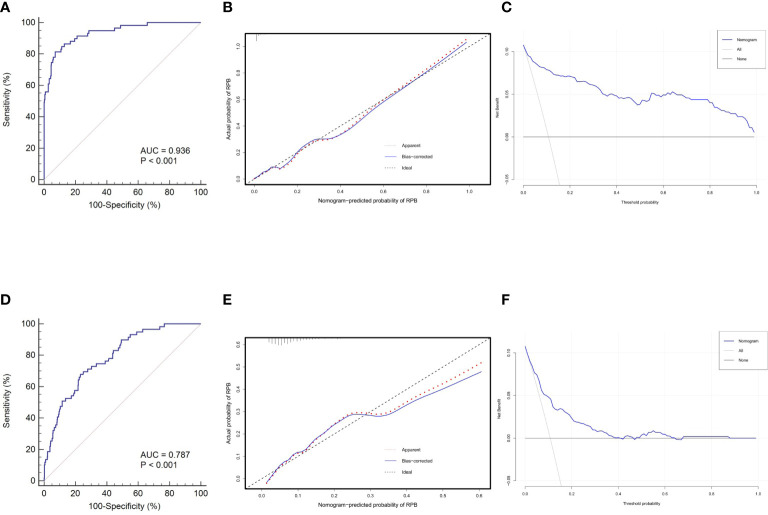
Nomogram validations in different cohorts. The ROC curve **(A)**, calibration plot **(B)** and DCA curve **(C)** reveal favorable discrimination, calibration and clinical net benefit in the training cohort. In the validation cohort, this model still shows acceptable discrimination **(D)**, calibration **(E)**, and clinical utility **(F)**, albeit with lower power than the training cohort. ROC, receiver operating characteristic; AUC,area under the curve; DCA, decision curve analysis.

The discrimination, calibration, and clinical utility for the developed nomogram were first evaluated in the training cohort. Following 1,000 bootstrapping, the stability of the model was tested to adjust the overfitting deviation. An adjusted C-index of 0.936 and a nonsignificant P value of the HL test (χ^2^ = 5.796, *P* = 0.670) indicated good discrimination and calibration power, as indicated in **Figures 5A, B**. Moreover, the result of DCA, which is displayed in **Figure 5C**, demonstrated that stratifying the RBP of OVCF patients using the nomogram would obtain clinical net benefits.

Subsequently, this model was independently tested in the validation cohort and found that it still revealed satisfied discrimination (C-index = 0.787) (**Figure 5D**) and calibration (χ^2^ = 8.327, *P* = 0.238) (**Figure 5E**). The DCA plot in **Figure 5F** suggested that although the clinical net benefit of the application in the validation cohort to predict the RBP risk was lower than in the training cohort, it still had good potential for clinical utility.

### Prediction accuracy comparison

**Figure 6:**
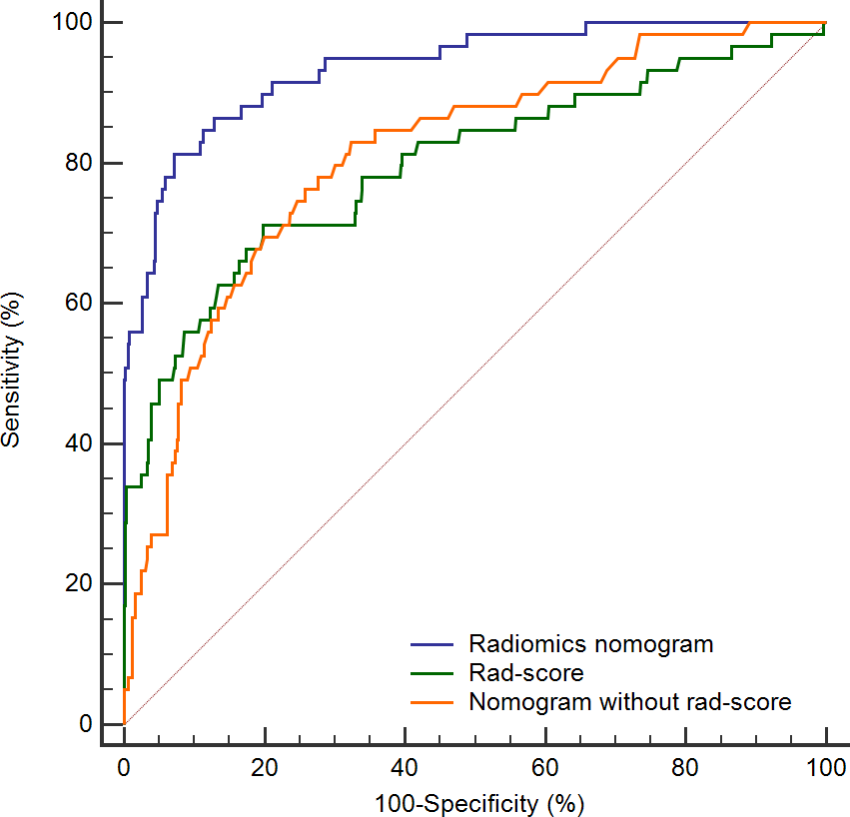
ROC curves for rad-score (green line), radiomics nomogram (blue line), and nomogram without rad-score (orange line). The AUC of radiomics nomogram is significantly higher than the remaining two curves (both *P* < 0.05). ROC, receiver operating characteristic; Rad-score, radiomics score; AUC, area under the curve.

To further highlight the complementary role of radiomics in prognostic prediction, we compared the predictive performance of the rad-score itself, and the nomograms with and without the rad-score. As shown in **Figure 6**, the prediction accuracies of the rad-score itself and the nomogram that only incorporated regular features were similar (AUC: 0.797 and 0.810, respectively), and both were significantly lower than the radiomics nomogram (AUC: 0.936, both *P* < 0.05). It implied that the model combining human-recognizable and computer-extracted features was more accurate for prognosis prediction.

## Discussion

Since postoperative RBP may significantly lower patient satisfaction with surgery, accurate preoperative estimation of the likelihood of RBP in OVCF patients contributes to improved outcomes and satisfaction of the vertebral augmentation. Thus, we built a nomogram combining the rad-score with regular features (BMD, IVC, and TLF injury) to preoperatively determine the likelihood of encountering RBP in OVCF patients. This model exhibited favorable discrimination, calibration and clinical utility in both the training and validation cohorts. Moreover, we proved that the radiomics nomogram achieved better performance than the rad-score and the nomogram only incorporated the regular features for the estimation of RBP risk.

Radiomics features are mathematically derived variables that have gained considerable popularity in the past 5 years because they indicate intra-region heterogeneity and assist the clinical diagnosis (19). The selected radiomics features offer a non-invasive, straightforward, and repeatable method to acquire microcosmic features (such as texture features) that fail to be evaluated by visual interpretation (20, 21). The performance of radiomics-based image analysis is equivalent or superior when compared to human interpretation. Its enormous potential in oncological imaging has been demonstrated by extensive radiomics evaluations (22). In skeletal diseases, although some studies have found that radiomics can evaluate the heterogeneity in femoral and spinal microarchitecture (23–25), compared with the number of published studies about oncologic radiomics, musculoskeletal radiomics investigations are less frequently reported (26). In the present study, we investigated, for the first time, the use of radiomics features to preoperatively predict RBP risk in OVCF patients receiving PKP. After reduction by LASSO regression, eight most relevant features with RBP were finally screened. However, the interpretation of the intricate associations between radiomics features and biological processes remains difficult (27). It is challenging to clearly explain specific radiomics parameters relate to pathophysiological changes, and the current solution is to develop a multi-feature strategy for result estimates (28). Thus, we developed and validated a combined nomogram that integrates regular features related to RBP with the rad-score for individualized estimation. According to the results, the rad-score performed similarly to the nomogram incorporating only regular features, and the combined nomogram achieved the best predictive performance. It implied that the radiomic features might improve diagnostic accuracy and efficiency by reducing the variability of subjective visual analyses and providing quantitative data (29), which has also been supported by several investigations (30, 31)

Over the past decade, an increasing number of studies have focused on the risk factors associated with RBP (9, 11, 12, 32). As revealed in our study, RBP was independently associated with BMD, IVC, and TLF injury. The impact of BMD on RBP is influenced by a number of factors. Patients with low BMD will experience insufficient bone cement dispersion and instability of the injured vertebra as a result of the absence of the typical trabecular structure in the vertebral body. Besides, patients with low BMD are at a higher risk of refractures, and there is a greater chance that surgical vertebral bodies or nearby vertebral bodies will fracture again (33, 34). The necrotic cavity formed by the IVC may lead to poor diffusion of the cement, which prevents a tight integration between the cement and the surrounding cancellous bone, thus causing instability of the fractured vertebrae and RBP after surgery (35, 36). The mechanism of TLF injury has not been well understood currently. The possible explanation is that the TLF is abundant in nerve endings, and the pain caused by TLF injury is easy to be ignored due to the severe pain of OVCF before surgery. While postoperative pain associated with fractures is relieved, pain due to TLF injury becomes more noticeable (11, 13). Although RBP-related influencing factors and underlying mechanisms have been reported, these published studies have failed to provide an applicable prediction model. Therefore, we integrated these independent correlates to assess the risk of RBP based on the nomogram, which has been accepted as a reliable tool for predicting individual risk (37).

The radiomics nomogram demonstrated strong predictive ability for RBP risk in OVCF patients, with satisfied discrimination and calibration. DCA indicated that this model improved the net benefit of preoperative RBP prediction. Even so, there are some limitations in this study. First, this is a single-center study using the same CT scanner designed for the convenience of image processing, although we have independently validated the model. Additional datasets from multiple medical centers are needed in future studies to test the reproducibility of the radiomics nomogram. Second, due to the practical situation in our hospital, only PKP performed by a unilateral approach was considered, which might introduce selection bias. However, no studies have indicated that the vertebral augmentation approach affects RBP. Third, considering the convenience of radiomics analysis, only patients with single-segment fractures were included in this study. Therefore, the model is only suitable for the prediction of patients with single-segment fractures. Finally, the translation of radiomics into clinical practice is still in its infancy. The reproducibility and validation of various radiomics techniques are currently not standard, and alterations in any step can affect the features and final output, implying barriers to clinical implementation.

## Conclusion

This study developed a nomogram including the rad-score and regular features of RBP for preoperative prediction of likelihood of RBP in OVCF patients, which was tested in multiple ways to indicate strong prediction performance. We believe the nomogram may serve as a potential tool to provide clinicians with more precise information for decision-making, patient education, and postoperative care, leading to improved surgical outcomes and satisfaction.

## Data availability statement

The raw data supporting the conclusions of this article will be made available by the authors, without undue reservation.

## Ethics statement

The studies involving human participants were reviewed and approved by Ruijin Hospital, Shanghai Jiao Tong University School of Medicine (2013-60). The patients/participants provided their written informed consent to participate in this study.

## Author contributions

CG and ZC contributed equally to this study and are co-first authors. Study design: CG, ZC, and XC. Data collection and analysis: ZC, YL, YZ, PC, and XC. Supervision: CG Statistics: CG, YL, and YZ. Manuscript writing: CG, ZC, and PC. Manuscript revision: CG, ZC. All authors contributed to the article and approved the submitted version.
