# Supplementary material for: Preoperative prediction of residual back pain after vertebral augmentation for osteoporotic vertebral compression fractures: Initial application of a radiomics score based nomogram
Source: Front Endocrinol (Lausanne). 2022 Dec 23;13:1093508. doi: 10.3389/fendo.2022.1093508 (PMC9816386; doi:10.3389/fendo.2022.1093508)
Supplement: Supplementary file 1 [file DataSheet_1.docx]

**Supplementary**

**Appendix 1**

Rad-score = 0.1076642-0.002212* gradient_glcm_ClusterProminence

+0.001113* squareroot_glcm_InverseVariance

+0.074552*wavelet-LLH_firstorder_Variance

+0.048543* wavelet-LHH_glszm_LargeAreaLowGrayLevelEmphasis

+0.003935* wavelet-HLL_firstorder_Kurtosis

+0.075954* wavelet-HLL_gldm_DependenceNonUniformityNormalized

+0.085155* wavelet-HLL_glszm_GrayLevelNonUniformityNormalized

+0.046348* wavelet-HLH_firstorder_Skewness
