# Supplementary material for: Preoperative prediction of residual back pain after vertebral augmentation for osteoporotic vertebral compression fractures: Initial application of a radiomics score based nomogram
Source: Front Endocrinol (Lausanne). 2022 Dec 23;13:1093508. doi: 10.3389/fendo.2022.1093508 (PMC9816386; doi:10.3389/fendo.2022.1093508)
Supplement: Supplementary file 2 [file DataSheet_2.docx]

**Supplementary**

**Table 1**. Basic characteristics and preoperative radiological parameters in the training and validation cohorts.

| Variable | | Training cohort (n=548) | Validation cohort (n=183) | *P* value |
| --- | --- | --- | --- | --- |
| Age, year | | 69.9 ± 3.9 | 71.1 ± 4.3 | 0.652^*^ |
| Gender, n(%) | Male | 124 (22.6%) | 40 (21.9%) | 0.829^$^ |
|  | Female | 424 (77.4%) | 143 (78.1%) |  |
| BMI, kg/m^2^ | | 23.8 (22.0 - 25.5) | 23.9 (21.9 - 25.7) | 0.362^#^ |
| Fracture position, n(%) | T4 - T10 | 57 (10.4%) | 15 (8.2%) | 0.580^$^ |
|  | T11 - L2 | 264 (48.2%) | 86 (47.0%) |  |
|  | L3 - L5 | 227 (41.4%) | 82 (44.8%) |  |
| Hypertension, n(%) | | 241 (44.0) | 83 (45.4%) | 0.745^$^ |
| Diabetes, n(%) | | 56 (10.2%) | 14 (7.7%) | 0.307^$^ |
| Smoking, n(%) | | 97 (17.7) | 22 (12.0%) | 0.072^$^ |
| BMD, T-score | | 3.14 ± 0.38 | 3.10 ± 0.34 | 0.091^*^ |
| Preoperative VAS, score | | 7 (6-8) | 7 (6-8) | 0.610^#^ |
| Preoperative ODI, score | | 43 (40-44) | 42 (40-44) | 0.185^#^ |
| Vertebral height loss (%) | | 34.2 ± 6.0 | 34.8 ± 6.2 | 0.270^*^ |
| Cobb angle, (°) | | 27.5 ± 4.9 | 28.0 ± 5.0 | 0.211^*^ |
| IVC, n(%) | | 47 (8.6%) | 18 (9.8%) | 0.604^$^ |
| TLF injury, n(%) | | 35 (6.4%) | 14 (7.7%) | 0.554^$^ |

Variables are expressed median (interquartile range) if they are not normally distributed. ^*^for independent sample t-test, ^$^for chi-square test, and ^#^for Mann-Whitney U test. BMI: body mass index, BMD: bone mineral density, VAS: visual analogue scale, ODI: oswestry disability index, Cobb angle: the angle between the superior and inferior endplate of the fractured level, TLF: thoracolumbar fascia, IVC: intravertebral cleft.
